# Mouse tracking reveals structure knowledge in the absence of model-based choice

**DOI:** 10.1038/s41467-020-15696-w

**Published:** 2020-04-20

**Authors:** Arkady Konovalov, Ian Krajbich

**Affiliations:** 1 Zurich Center for Neuroeconomics, Department of Economics, University of Zurich, Blümlisalpstrasse 10 8006 Zurich, Switzerland; 2 Department of Economics, The Ohio State University, 1945 North High Street, Columbus, OH 43210 USA; 3 Department of Psychology, The Ohio State University, 1827 Neil Avenue, Columbus, OH 43210 USA

**Keywords:** Learning algorithms, Operant learning, Reward, Human behaviour

## Abstract

Converging evidence has demonstrated that humans exhibit two distinct strategies when learning in complex environments. One is model-free learning, i.e., simple reinforcement of rewarded actions, and the other is model-based learning, which considers the structure of the environment. Recent work has argued that people exhibit little model-based behavior unless it leads to higher rewards. Here we use mouse tracking to study model-based learning in stochastic and deterministic (pattern-based) environments of varying difficulty. In both tasks participants’ mouse movements reveal that they learned the structures of their environments, despite the fact that standard behavior-based estimates suggested no such learning in the stochastic task. Thus, we argue that mouse tracking can reveal whether subjects have structure knowledge, which is necessary but not sufficient for model-based choice.

## Abstract (web summary)

Mouse tracking can reveal people’s subjective beliefs and whether they understand the structure of a task. These data demonstrate that people often do not use this information to make good choices.

## Introduction

A central question in the study of behavior is to what extent decisions are driven by top-down goals versus bottom-up reward associations. To understand this question, researchers have used multi-stage decision tasks where one’s decision in the first stage affects the options available at later stages. It has been argued that such tasks require the decision maker to understand the task structure and plan ahead, in order to optimize performance. Such behavior is referred to as ‘model-based’ learning^1–21^, and is typically understood as the ability to use the structure of the environment in order to reach goals and receive rewards. Individual inclination to use this type of strategy has been linked to goal-based behavior^22,23^, cognitive control^5^, slower habit formation^16^, declarative memory^24^, higher extraversion^6^, and lower alcohol dependence^25^.

However, while the initial studies of model-based behavior involved a now popular two-stage Markov decision task with stochastic action-state contingencies^1,17,23,26,27^, recent evidence suggests that model-based strategies do not typically lead to higher rewards in these probabilistic tasks. It is therefore unclear whether a lack of model-based behavior reflects an inability to learn the structure of the environment or simply indifference towards the model-based strategy.

One approach to this problem has been to devise new tasks where it is beneficial to use model-based strategies. For instance, some have argued that tasks with deterministic relationships between actions and outcomes might be better ways to study model-based behavior^14,21,28,29^. However, it is unclear to what extent learning in deterministic environments relates to learning in stochastic environments.

A second approach is to investigate the stochastic task more thoroughly. The stochastic task remains a standard for measuring model-based behavior, and has been used in many studies in both psychology and neuroscience^8^. It is therefore important to better understand what this task actually measures, using data beyond subjects’ choices. For instance, previous eye-tracking work has demonstrated that model-based strategies have distinct gaze signatures^17^. Recent research in other decision-making domains has highlighted the usefulness of studying peoples’ mouse trajectories in computer-based tasks. This research has focused on using the mouse trajectories to infer how strongly decision-makers favor their chosen options^30–36^. We reasoned that it should be possible to similarly use mouse trajectories to infer how strongly decision-makers expect particular outcomes.

In this study, we set out to investigate both of these approaches. First, we sought to identify whether the degree of model-based behavior at the individual level is consistent across different types of environments, using a two-stage task that allows for both stochastic and deterministic transitions within the same paradigm. Second, we used mouse tracking in both types of tasks to try to detect whether subjects were learning the structures (exhibiting structure knowledge), despite not necessarily using that information to change their behavior (being model-based in their choices). We employed a new study design that uses the cursor position as a measure of subjects’ action-state contingency beliefs and, in general, their structure knowledge.

**Fig. 1:**
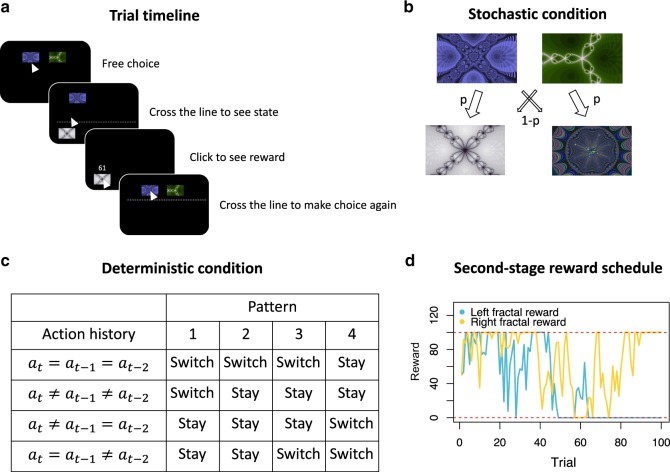
**Experimental task and design**. **a** Choice-trial timeline. Subjects chose between the two top fractals (first stage). To reveal the outcome (second stage), subjects needed to move the mouse below an invisible line on the screen (here shown with a dotted line). To receive the resulting reward, subjects clicked on the revealed bottom fractal. To begin the next trial, subjects moved the mouse back up above another invisible line. The positions of all fractals were fixed from trial to trial within a block. **b** In the stochastic condition, one of the top fractals is more likely to lead to one of the bottom fractals (common transition), and the probabilities are reversed for the other pair. **c** In the deterministic condition, the switch between the bottom fractals from trial to trial is determined by the choices of the top fractals in the three previous trials. Here we show the four deterministic patterns used in the experiment. $a_t$ is the top choice in trial $t$; the action history column describes the four possible top-choice histories. Each pattern (columns 1–4) is a set of outcomes corresponding to one of these histories. These outcomes include either stay (the same bottom fractal appears on this trial as in the previous trial) or switch (the bottom fractal switches from the previous trial). See Supplementary Information for a detailed description of the patterns. **d** Example of the second stage reward schedule: each bottom fractal had an independent drifting reward following a Gaussian random walk, with rewards restricted to the interval between 0 and 100. Each subject had independent, randomly generated reward schedules. The fractals pictured here are for illustration only; the originals are available in the OSF repository. Source data are provided as a Source Data file.

The new two-stage learning task used a single screen to display both the first and second stages (the structure of transitions between the states, however, still remains hidden to the subjects). As in many previous experiments, subjects chose between two stimuli (fractals) in the first stage, and these choices determined which of two second-stage fractals would appear. Unlike other studies, the second stage was not revealed immediately, or on a separate screen. Instead, subjects had to move the cursor below an invisible line to see the second-stage fractal (Fig. 1a, see Methods). Because subjects are naturally motivated to finish tasks quickly, they had an incentive to move their cursor to the side of the screen where they expected the second-stage fractal to appear. This allowed us to measure subjects’ beliefs about the locations of the fractals and to determine whether they had learned the transition structure between the first-stage choices and the second-stage outcomes.

Within the same paradigm, we used both stochastic transition structures (Fig. 1b) and deterministic transition structures (Fig. 1c). In the stochastic task one of the first-stage fractals was more likely to lead to one of the second-stage fractals; this is referred to as a common transition (as opposed to a rare transition). In the deterministic task there were patterns, where a short sequence of first-stage choices deterministically determined the second-stage fractal.

We implemented several recent suggestions to improve the correlation between the degree of model-based behavior and reward rate: using different reward amounts instead of different probabilities of a fixed reward, a wide range of rewards, large changes in mean reward from trial to trial, and no choice in the second-stage^28^. Nevertheless, in the stochastic task, subjects’ earnings were still uncorrelated with the degree to which they were model-based in their choices. Indeed, when we simulated model-based agents in this task, it took thousands of trials to generate a significant correlation between model-based behavior and reward rate. Meanwhile, in the deterministic task, model-based choice yields higher rewards even with a relatively low number of trials (100). This experiment allowed us to directly compare subjects’ behavior in stochastic and deterministic environments and to study the information contained in mouse trajectories in cases where subjects were and were not incentivized to use model-based behavior.

To preview the results, we find that mouse tracking can reveal individuals’ subjective beliefs and we demonstrate that even though individuals learn the task structure, their choices do not necessarily become model-based.

## Results

In the stochastic task, one can qualitatively distinguish model-based and model-free choices by comparing behavior after common and rare transitions^1^. To demonstrate this, we simulated purely model-free and model-based agents using the standard models (see Methods). The simulations use values of the learning rate and temperature parameters close to the median values in the experiment (α = 0.7, β = 0.1), although the qualitative difference between model-based and model-free behavior does not depend on the specific values of these parameters.

**Fig. 2:**
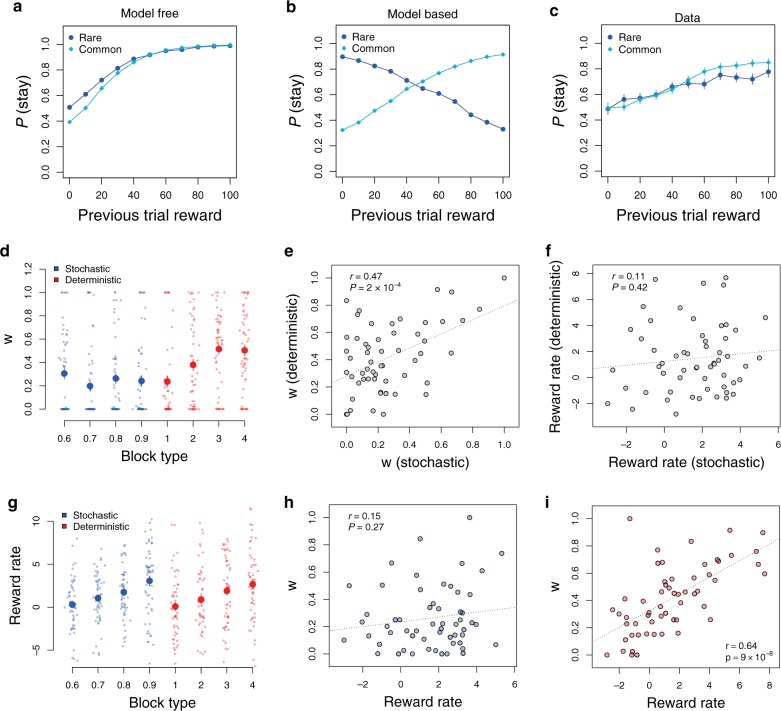
**Simulations and behavioral results**. **a** Example simulation of a purely model-free agent (*w* = 0) in the stochastic transition task: the probability to stay with the same first-stage choice increases with the previous trial’s reward, but does not depend on the previous transition (common (dark blue circles) or rare (light blue diamonds)). **b** Example simulation of a purely model-based agent (*w* = 1) in the stochastic transition task: the probability to stay with the same first-stage choice decreases with the previous trial’s reward if the previous transition was rare. **c** Probability to stay with the same first-stage option in the stochastic transition task: subjects display very little model-based behavior (*N* = 57 individual subjects). **d** Model-based weight *w* by condition (block type): 0.6–0.9 correspond to the common transition probability (stochastic condition, in blue), and 1–4 denote the pattern type (deterministic condition, in red). **e** Correlation in model-based weight *w* between stochastic and deterministic conditions. The weights are calculated as the averages of block-wise estimates. **f** Correlation in reward rate between stochastic and deterministic conditions. **g** Reward rate by conditions (block type) and pattern difficulty (*N* = 57 individual subjects). **h**–**i** Correlation between the model-based *w* and reward rate in the stochastic (**h**) and deterministic (**i**) conditions. All correlation plots show Pearson correlations, each individual point is one subject, and dotted lines indicate linear regression fits. In bar plots: dots denote individual subjects, error bars denote s.e.m. at the subject level. Source data are provided as a Source Data file.

For the model-free case, the first-stage choice is more likely to be repeated if the previous trial yielded a high reward, regardless of the transition type (Fig. 2a). In contrast, for the model-based case, the probability of repeating the same first-stage choice after a high reward depends critically on whether the transition was common or rare. After a rare transition, a high reward should encourage the subject to switch in the next trial, in order to increase their chance of reaching that high-reward state again (Fig. 2b).

The data from the experiment show that subjects were generally not model-based in their choices (Fig. 2c). We confirmed this with a mixed-effects logit regression of the decision to stay with the same first-stage option as a function of the reward in the previous trial, for rare transitions (using R packages lme4 and lmerTest). For model-based behavior we expect this relationship to be negative; in our data it is positive: *β* = 0.02, *z* = 6.48, *p* = $9 \cdot 10^{ - 11}$. There was some degree of individual variability: only 8 subjects had negative regression coefficients (more model-based behavior), while the other 49 subjects had positive coefficients (more model-free behavior).

To allow for comparisons between the two tasks, we also modeled subjects’ behavior using the same standard model-based reinforcement learning we used for the simulations (see Methods), determining the degree to which they used knowledge of the structure to make their first-stage choices (characterized by a weight parameter *w*, where *w* = 0 indicates pure model-free behavior and *w* = 1 indicates pure model-based behavior). This model provided a good fit to the data, explaining (on average, across subjects), 73% of subjects’ choices. Across subjects, the model-based weight *w* was 0.24 (sd = 0.23) in the stochastic task and 0.41 (sd = 0.25) in the deterministic task (the difference is significant; two-sided *t*-test, t(56) = 4.63, *p* = $2 \cdot 10^{ - 5}$, Fig. 2d). There was no significant time trend in *w* across the blocks (mixed effects regression, stochastic task: *p* = 0.61, deterministic task: *p* = 0.59).

As expected, for the stochastic task, the regression measure of model-based behavior was correlated with *w* from the full model estimation (Pearson’s *r* = −0.53, t(55) = −4.6, *p* = $2 \cdot 10^{ - 5}$). The simulations above revealed that a positive coefficient corresponds to *w* < 0.5 (so $\beta \, = \,0\, \Leftrightarrow \,w\, = \,0.5$), and indeed 47 out of 57 subjects had *w* < 0.5.

It has been suggested that both stochastic and deterministic transition structures can be used to study model-based behavior. However, since these types of transitions could lead to very different representations of the environment, it was unclear whether being model-based in one setting would correlate with being model-based in the other. We found that individual *w* in these two conditions, calculated as averages of block-wise estimates, were indeed correlated (Fig. 2e, Pearson’s *r* = 0.47, t(55) = 4.00, *p* = $2 \cdot 10^{ - 4}$).

Despite the correlation in *w* between the two tasks, there was no correlation in the reward rates between the tasks (Pearson’s r = 0.11, t(55) = 0.81, *p* = 0.42, Fig. 2f). Again, this is consistent with the idea that in the two-stage task with stochastic transitions, reward rate is not correlated with the index of model-based behavior^28^. The reason is that the model-based strategy does not help participants earn higher rewards^15^. This was also the case in our data; there was no correlation between *w* and reward rate in the stochastic tasks (Pearson’s *r* = 0.15, t(55) = 1.11, *p* = 0.27, Fig. 2h). In comparison, reward rate had a strong positive correlation with *w* in the deterministic task (Pearson’s *r* = 0.64, t(55) = 6.16, *p* = $9 \cdot 10^{ - 8}$, Fig. 2i).

We next sought to test whether *w* might reflect the difficulty of the task. We hypothesized that participants would show more model-based choice when it was easier (i.e., less costly) to learn the transition structure. Starting with the deterministic task, we indeed observed higher values of *w* for easier patterns (as indexed by reward rate) (Fig. 2d, mixed effects regression, $\beta = 0.09$ (s.e. 0.02), t(56) = 4.58, *p* = $3 \cdot 10^{ - 5}$). However, in the stochastic task there was no change in *w* as the common transition probability increased from 0.6 to 0.9 (Fig. 2d, mixed effects regression, $\beta = - 0.12$ (s.e. 0.2), t(55) = −0.6, *p* = 0.54). Reward rate did increase with the common transition probability (mixed effects regression, $\beta = 8.9$ (s.e. 2.2), t(64) = 4.6, *p* = $8 \cdot 10^{ - 6}$, Fig. 2g) but this would also occur for a pure model-free learner (as confirmed by a simple simulation of a model-free subject).

**Fig. 3:**
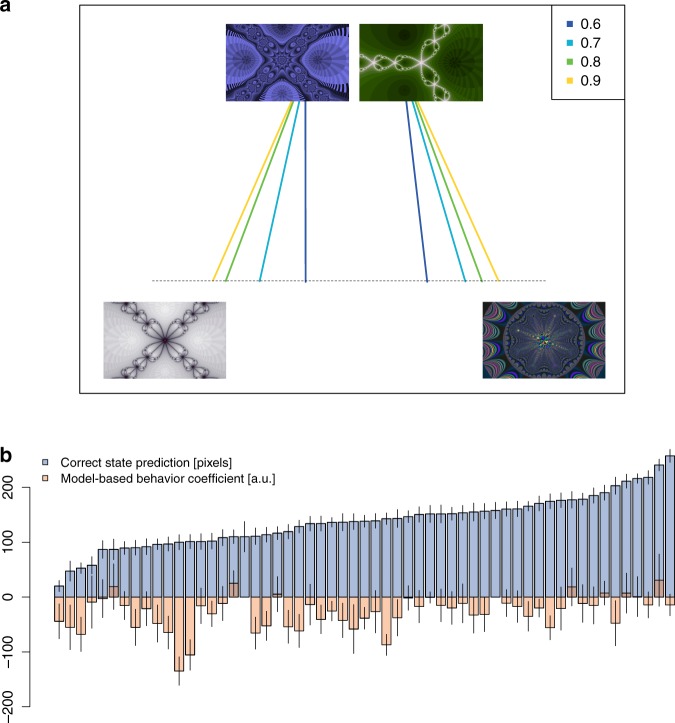
**Mouse-tracking results: stochastic task**. **a** Ballistic mouse trajectories in the stochastic task. Each colored line represents the average cursor path between a top fractal and a bottom fractal for a specific transition probability (yellow = 0.9, green = 0.8, light blue = 0.7, dark blue = 0.6). For illustration, we only present the paths between fractals on the same side of the screen, in cases where subjects’ predictions were correct. We only measure the cursor’s starting point and the point at which it crosses the line, connecting those two points with a straight line. **b** Individual subject estimates in the stochastic task. In blue: distance towards the correct (common transition) side of the screen (in pixels), in increasing order (error bars represent s.e.m. based on *N* = 400 trials for each subject). In orange: inverted regression-coefficient measure of model-based behavior from Fig. 2a-c (in arbitrary units, with positive values denoting more model-based behavior, errors bars represent standard error of the regression coefficient of each individual subject based on *N* = 400 trials). Source data are provided as a Source Data file.

We next turned to subjects’ mouse movements as a direct measure of whether they had learned the structure of the action-state transitions, i.e., the model-based knowledge. If participants were motivated to complete the task quickly^37^, then those who learned the transition structures might anticipate the location of the next second-stage fractal and move their mouse in that direction before actually seeing the fractal. We used a mouse-tracking measure of the pixel distance between the vertical midline and the mouse cursor’s crossing point on the invisible horizontal line, to provide a separate estimate of subjects’ knowledge of the task structure (Fig. 3a, Methods). This measure was positive if the cursor was on the correct side of the screen, i.e., towards the common transition in the stochastic task and the coming state in the deterministic task.

**Fig. 4:**
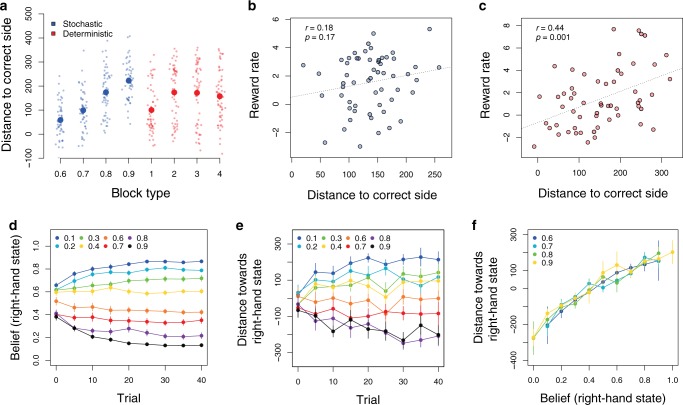
**Mouse-tracking results**. **a** Distance towards the correct bottom fractal by condition and difficulty. **b**–**c** Correlation between reward rate and mouse-tracking measure of learning, split by condition: stochastic (**b**) and deterministic (**c**). **d** Stochastic task: evolution of the posterior Bayesian probability of revealing the bottom right-hand fractal, conditional on the top left-hand fractal choice, split by condition, for the first 40 trials of each block (in bins of size 5). **e** Stochastic task: evolution of the mouse cursor towards the right-hand side of the screen, split by condition, for the first 40 trials of each block (in bins of size 5). **f** Cursor distance as a function of Bayesian belief (in bins of size 0.1), by condition. All correlation plots show Pearson correlations, each individual point is one subject, and dotted lines indicate linear regression fits. In **a**–**c**: dots denote individual subjects, in all plots: error bars denote s.e.m. at the subject level (*N* = 57). Source data are provided as a Source Data file.

In line with the behavioral results and our mouse-tracking hypothesis, in the deterministic task, subjects’ cursors were significantly different from zero for every pattern (two-sided *t*-tests, Cohen’s *d* = 1, t(51) = 7.2, Cohen’s *d* = 1.48, t(55) = 10.8, Cohen’s *d* = 1.38, t(54) = 10.2, Cohen’s *d* = 1.45, t(55) = 10.8, all *p* < 0.0001), and farther in the correct direction (i.e., towards where the fractal actually appeared) for easier patterns (Fig. 4a; mixed effects regression, $\beta = 16.6$ pixels per pattern (s.e. 5.7), t(51) = 2.8, *p* = 0.006). Moreover, mouse movements were significantly correlated with *w* for the deterministic task (*r* = 0.57, t(55) = 5.15, *p* = 4 $\cdot 10^{ - 6}$, Supplementary Fig. 1). Thus, mouse position did appear to track subjects’ learning.

Turning to the stochastic task we also observed clear evidence of learning of the transition structure; subjects’ cursors were significantly on the correct side (i.e., towards where the fractal was most likely to appear) for all values of the common transition probability (two-sided *t*-tests, Cohen’s *d* = 0.82, t(52) = 5.95, Cohen’s *d* = 1.24, t(54) = 9.2, Cohen’s *d* = 1.82, t(52) = 13.3, Cohen’s *d* = 2.87, t(54) = 21.3, all *p* < 0.001) and closer to the correct fractal as the common transition probability increased (Fig. 3a, mixed effect regression $\beta = 56.4$ pixels per 0.1 increase in transition probability (s.e. = 48.8), t(64) = 11.6, *p* = $10^{ - 16}$).

For the stochastic task we ordered subjects by their mouse-tracking measure of structure knowledge (Fig. 3b). Statistically, nearly every subject (56 out of 57) had a significantly positive effect (all *p* < 0.01, t(399) > 2.6, *t*-test against 0). In addition, we focused on the blocks showing no model-based behavior. Specifically, we selected the blocks where *w* was estimated at 0 (51% of the stochastic task data) and the blocks where the interaction between common/rare transition and previous trial reward was negative or equal to 0 (42% of the stochastic task data). In both cases, almost all subjects showed a significant mouse-tracking measure of the transition-structure knowledge (two-sided *t*-test; *p* = 10^−6^, t(49) = 12.6, and t(46) = 11.3; Supplementary Fig. 2).

Clearly our subjects learned the transition structure. This result indicates that *w* does not necessarily reflect knowledge of the transition model; these subjects clearly knew the transition structure, they simply did not make their choices in a model-based way.

There were significant correlations between the individual mouse-tracking measures of structure knowledge and the regression measures of model-based choice (Fig. 3b, Pearson’s *r* = −0.36, t(55) = −2.84, *p* = 0.006), as well as with *w* (Pearson’s *r* = 0.3, t(55) = 2.3, *p* = 0.025, Supplementary Fig. 1). Thus, as one would expect, there is a positive relationship between structure knowledge and model-based choice.

Turning to reward rates, in the stochastic condition our mouse-tracking measure of structure learning did not correlate with individual reward rate (Fig. 4b, Pearson’s *r* = 0.18, t(55) = 1.39, *p* = 0.17), while in the deterministic condition it did (Fig. 4c, Pearson’s *r* = 0.44, t(55) = 3.66, *p* = 0.0002). These results indicate that when model-based behavior is useful for earning larger rewards, our mouse-tracking measure reliably correlates with it.

Interestingly, we observed a weak but significant correlation between the mouse movements in the stochastic and deterministic condition (Pearson’s *r* = 0.26, t(55) = 2.02, *p* = 0.048). This suggests some overlap in the ability to learn the two types of structures.

Finally, we analyzed the trial-by-trial mouse positions in the stochastic task, where it is possible to derive subjective beliefs (conditional action-state probabilities) as Bayesian posteriors assuming a Beta-Bernoulli distribution^9,17^. These analyses revealed a striking similarity between the evolution of Bayesian beliefs and subjects’ mouse movements across trials (Fig. 4d-e). In other words, the expected strength of a subject’s belief that their action will lead to the fractal on the right predicts how far their mouse will move to the right (Fig. 4f). Here, we aggregated across all trials, both first-stage options (left and right), and all probabilistic action-state contingencies. The correlation was (Pearson’s) *r* = 0.69, t(55) = 7.1, *p* = $3 \cdot 10^{ - 9}$ (subject level, all conditions pooled), which is also confirmed by a linear mixed-effects regression of the distance towards the right-hand state as a function of the belief that the right-hand state is coming, treating subjects as random effects ($\beta = 0.03$ (s.d. = 0.003), t(54) = 10.3, *p* = $2 \cdot 10^{ - 14}$). Notably, we observed no corresponding change in model-based choice across trials (see Supplementary Fig. 3).

On trials immediately following rare transitions, subjects’ mouse cursors did not display the typical deviation towards the correct side, instead crossing near the middle of the screen, or in some cases on the wrong side. This suggests one reason why subjects may not have been choosing in a model-based way in the task; whenever there was a rare transition their beliefs appear to have temporarily deviated from the ideal. However, subjects’ mouse movements quickly (within ~2 trials) realigned with the expected curve (see Supplementary Fig. 4).

Finally, we also examined trials with rare transitions that led to a large (>50) reward (6% of trials overall). In such cases, a model-based subject should often switch on the following trial, while a model-free subject should stay. Based on the mouse trajectory during the rare-transition trial, we found that subjects were indeed significantly more likely to switch after a correct mouse trajectory than after an incorrect trajectory (logistic mixed effects regression, $\beta = - 0.53$ (s.d. = 0.2), *z* = 2.64, *p* = 0.008). However, in both cases subjects were more likely to stay than to switch, confirming again that subjects were more model-based in their mouse movements than in their choices (Supplementary Fig. 5).

## Discussion

Our results demonstrate that in a classic two-stage learning task with stochastic transition structures, subjects’ behavior does not necessarily reflect their knowledge of the structure, as revealed by mouse tracking. Subjects appear to often know exactly which second-stage state is coming but do not use this information when making their first-stage choices. These results suggest that the absence of model-based decisions does not imply that an individual has not built a model of the environment: if this knowledge is not useful for receiving larger rewards, they might choose not to use it. One can think of model-based behavior as consisting of two necessary-but-not-sufficient components: structure knowledge and attribution. We use the mouse-tracking data to identify the structure knowledge component and show that it is often present in the absence of the attribution component (or high *w*, i.e., model-based behavior).

This observation is consistent with other work finding that at the end of the experiment, most subjects can describe the transition structure^38^. Our results confirm that this is not simply due to reflection at the end of the experiment, but is indeed knowledge that subjects have throughout the task. In the deterministic case, the model-based strategy yields a clear reward advantage, making it more attractive to learn the structure and use that knowledge to earn larger rewards, while the purely model-free strategy does not perform better than chance.

Our results also show that in the traditional stochastic task, despite subjects knowing the transition structure, they do not seem to utilize it for model-based reinforcement. Understanding why this proper credit assignment does not occur is a critical open question. As other studies show, in this probabilistic task there is little benefit to employing model-based behavior, so if it is costly to use model-based knowledge to guide choices, subjects may not actively employ it. So, one way to frame our findings is that it is not costly to learn the model-based structure, but it is costly to use that structure to guide behavior.

It is possible that the mouse movements in the stochastic task could be learned in a model-free way. However, subjects would still possess the knowledge of which first-stage actions lead to which second-stage states. The puzzle is why they wouldn’t use that knowledge to alter their choice behavior. One can also argue against model-free learning of the mouse movements since this would not be possible (or at least much more complicated) in the deterministic task, where the same first-stage choice leads to different second-stage states depending on the prior history. In addition, the pattern in Supplementary Fig. 4 indicates immediate dampening/resetting of the mouse-movement—belief association after a rare transition, followed by gradual (but quick) recovery, which is inconsistent with a simple RL mechanism.

The results in Supplementary Fig. 4 also reveal that subjects’ inability to behave in a model-based way may be due not just to attribution, but also to temporary forgetting of the structure knowledge after rare transitions, which is precisely when model-based behavior is identifiable. Thus, an inability to represent a stable transition structure may be at the root of the problem. This could be another manifestation of the hot-hand fallacy^39^.

Although others have argued for a relationship between payoff-relevance and the model-based index^28^, here we observed clear evidence that the individual model-based indices are consistent between the probabilistic and deterministic tasks, while evidence for consistent structure knowledge was considerably weaker. This suggests that acting on model-based information may be a more stable individual trait than the ability to learn the structure itself. Thus, the standard stochastic task may still be useful for evaluating the natural tendency to behave in a model-based way, even if model-based behavior is not incentivized.

Finally, our results demonstrate a simple but powerful method of mouse tracking^40^ that does not require tracing the entire mouse trajectory but instead its location at a single point in time^41^. The approach relies on a similar mechanism to predictive gaze^42^, but does not necessitate the use of eye-tracking equipment. This makes the technique easy to use and useful for evaluating what subjects believe in a simple non-invasive way that could be applied to any task involving beliefs. Future research could attempt to use mouse-tracking data at the trial level, to provide more direct measures of subjects’ latent beliefs. This would allow researchers to track changes in beliefs in a more precise way than using discrete choice data.

## Methods

### Participants

We recruited 58 adult subjects (20 female) from the Department of Economics undergraduate subject pool at the Ohio State University. We paid each subject based on overall performance in the task, with subjects earning $13.3 on average, including $5 as a show-up fee. We determined the target sample size aiming to estimate a significant correlation between an individual mouse-tracking measure and model-based index assuming a Pearson correlation coefficient of 0.5, 0.01 significance level, and 90% power, which resulted in a minimal sample of 52 subjects. We invited 60 subjects for two 30-person sessions. Out of 58 subjects who participated, we excluded one subject who failed to complete the task in reasonable time, leaving 57 subjects for all the analyses. The Ohio State University Internal Review Board approved the experiment, and all subjects provided written informed consent.

### Task

We used a modified version of the two-stage task commonly used to estimate the index of individual model-based behavior^1,29^, implementing a series of recent recommendations that improve the estimation of the parameter of interest^28^. We used Psychtoolbox in MATLAB (Mathworks) to present the stimuli and record mouse-tracking data. For each subject, we recorded the position of the mouse cursor on the screen at a rate of 1000 Hz.

Each trial had two stages. Unlike previous experiments in the literature, we presented all the states of the two-stage task on the same screen to allow for mouse tracking. In the first stage, subjects chose one of two fractals (let us label them A1 and A2) presented at the top of the screen (Fig. 1a), with no time restriction. Each choice could lead to one of the two separate states, represented by another pair of fractals (let us label them B and C), displayed in the bottom left and the bottom right corners of the screen. After a subject clicked on one of the first-stage fractals, the new state was not immediately revealed. To see the outcome, the subject had to move the mouse below an invisible line located 70% of the way to the bottom of the screen (Fig. 1a). Once the mouse cursor crossed the invisible line, the second-stage fractal appeared on the screen and the first-stage fractals disappeared. In the second stage there was no choice: the subject just had to click on the fractal to reveal the reward (again, with no time restriction). Once the reward was revealed, the subject had to move the cursor above another invisible line located 70% of the way back to the top of the screen, to reveal the first-stage fractals for the next trial (implying a self-paced intertrial interval (ITI)). The left/right positions of the all the fractals remained fixed throughout each block of trials.

### Experiment design

Each subject completed 8 blocks of the experiment, with each block consisting of 100 trials. Each block used a completely new set of fractals. Across the blocks, we varied the type of structural relationship between the first-stage choices and second-stage states.

Four blocks had the standard^1^ stochastic relationship, where each of the fractals A1 and A2 was more likely to lead to one of the two bottom fractals B and C (Fig. 1b). For instance, A1 would lead to B with probability 0.6, and to C with probability 0.4. For A2, these probabilities were reversed. We varied the common transition probability between the blocks, using the values 0.6, 0.7, 0.8, and 0.9. Within each subject, we randomly counterbalanced whether the right or left bottom fractal was more common for the left or the right top fractal. There was no evidence for a side bias across all conditions (t(56) = 0.98, *p* = 0.32) nor within any individual condition (*p* > 0.15).

The other four blocks had deterministic relationships that depended on the first-stage choice in previous trials (Fig. 1b). Specifically, we considered any choice history two trials back. Given only two options, there are four possible histories: same option chosen three times in a row (e.g., A1, A1, A1); different option chosen each time (e.g., A1, A2, A1); same option chosen one trial back, but not two trials back (e.g., A1, A1, A2); a different option chosen one trial back and two trials back (e.g., A1, A2, A2). We defined the possible outcome of these histories as repeating/switching the second-stage state on the current trial (from B to C or vice versa). Four possible histories and two possible outcomes produced 16 potential transition structures (or patterns), from which we selected four non-trivial ones. If the subject was able to figure out the hidden transition pattern, he or she could deterministically reach one of the desired bottom fractals by applying a specific first-stage choice sequence. For a detailed description of the four transition patterns please see the Supplementary Information. As an example, for the easiest pattern (pattern 4) the rule looked as follows: to get fractal B, the subject needed to choose the same fractal (A1 or A2) on every trial, while constant alternating between A1 and A2 always led to fractal C.

We presented all eight blocks in random order. Before each block, we indicated the type of the transition structure to the subjects. To avoid belief spillover and excessive experimentation, we instructed them, following the standard protocol, that one type of block had a random transition structure, with one of the top (first-stage) fractals being commonly associated with one of the bottom (second-stage) fractals, while the other type of block had a more complex transition pattern that they needed to figure out on their own.

Within each block, both bottom fractals had independent reward distributions (Fig. 1d). Mean rewards for each fractal drifted between 0 and 100 points according to a normal distribution with a standard deviation of 20, and each realized payoff had added normally distributed noise of mean 0 and standard deviation of 20; this was done to ensure that learning rates below 1 were optimal to succeed in the task^43^. At the end of the experiment, we converted the sum of all points earned in all blocks into each subject’s USD payoff.

### Computational modeling

We used the following standard model combining TD(1) (temporal difference) model-free learning and model-based learning to fit subjects’ choices and estimate the model-based index^1,17,23,27,28^. We assumed that the value of the chosen bottom fractal is updated using the model-free Rescorla-Wagner rule:

1 $$v_t = v_{t - 1} + \alpha \left( {r_t - v_{t - 1}} \right),$$

where $v_t$ is the value of the bottom fractal on trial *t* or *t*−1, *r*_*t*_ is the reward on trial *t*, and $\alpha$ is the learning rate. The model-free Q-value of each of the chosen top fractals is updated in a similar fashion (using a TD(1) update):

2 $$q_{{t}}^{{\mathrm{MF}}} = \left( {1 - \alpha } \right) \cdot q_{{{t}} - 1}^{{\mathrm{MF}}} + \alpha \cdot r_{{t}},$$

where $q_t$ is the value of the bottom fractal on trial *t* or $t - 1$, and *r*_*t*_ is the reward on trial *t*. For the sake of simplicity, we assumed that this value is updated with the same learning rate $\alpha$; the results are similar using two separate learning rates.

In addition, we assigned a model-based *Q*-value to the top fractal choice. In the stochastic condition, this value was equal to the expected value of the choice: $q_t^{{\mathrm{MB}}} = p_{\mathrm{L}}v_t^{\mathrm{L}} + p_{\mathrm{R}}v_t^{\mathrm{R}}$, where $p_i$ represent the true probabilities of getting to the left (L) or right (R) bottom fractals after choosing the specific option, and $v_t^i$ are the cached model-free values of the bottom fractals. In the deterministic condition, since the next bottom fractal was uniquely defined from the underlying pattern based on the previous history of top fractal choices, the model-based value of each top fractal was simply equal to the cached value of the bottom fractal that would appear (according to the pattern) if that top fractal was chosen.

In the final step of the model, we used the standard hybrid combination of the model-free and model-based *Q*-values:

3 $$q_t^{{\mathrm{HYB}}} = w \cdot q_t^{{\mathrm{MB}}} + \left( {1 - w} \right) \cdot q_t^{{\mathrm{MF}}},$$

where *w* is the weight index reflecting the degree of model-based behavior. We used the difference of *Q*-values for the top fractals as an input in a standard logistic choice model with a temperature parameter $\beta$.

To allow for greater parameter flexibility, we fit this model with three free parameters (*α*, *β*, *w*) to each separate 100-trial block using maximum likelihood estimation (MLE). Since *w* is our main parameter of interest, for all analyses we excluded 27 blocks where *w* could not be identified (about 6% of the data). This affected 17 subjects, with a maximum of 3 out of 8 blocks excluded per subject.

In addition, we explored several alternative models from the literature: a purely model-free learner (TD(1), *w* = 0), a TD(0)-hybrid model, a TD(1)-hybrid model including a perseverance parameter, a TD(λ)-hybrid model including an eligibility trace λ, and the TD(λ) model including the perseverance parameter, and including both of these last two parameters (see Supplementary Information for the detailed descriptions of the models). Although more complex models provide an improvement in fit for some subjects, in our case the average Bayesian information criterion (BIC) value for these models was worse than the simple TD(1) model, albeit by a small margin (see Supplementary Fig. 6). Given the lack of meaningful improvement with these models, we opted for the simplest model variant. Importantly, the main results related to the model fits do not depend on the choice of the model (see Supplementary Fig. 7).

### Measures of interest

In our analyses, we focused on three individual-level variables:

#### Reward rate

behavioral measure of performance. Since each subject has randomly drawn reward distributions, following previous literature, we normalized received rewards by simply subtracting the average of the two empirical reward distributions within each block from the total reward received by the subject in that block^28^.

#### Model-based weight *w*

a computational measure of model-based behavior. It is the standard index of individual model-based behavior in the literature^1^. Since we computed one weight per block, for cross-subject analyses we averaged these weights across relevant blocks. The results are similar using the median *w*.

#### Distance to the correct state side

mouse-tracking measure of model-based behavior. Since the resulting bottom fractal was only revealed after the mouse cursor crossed an invisible line on the lower part of the screen, we used the horizontal coordinate of the point where the cursor crossed the line as a measure of belief about the specific (left or right) fractal to be revealed on that trial. As model-based individuals should be tracking the environmental structure, they should be more likely to move the cursor to the side where the fractal will appear. Our specific measure was the absolute pixel distance between the point where the cursor crossed the invisible line and the midpoint of the line, if the cursor was on the correct side of the screen (in the stochastic task: where the common transition state should have appeared; in the deterministic task: where the state defined by the pattern was going to appear), and the negative of this distance if the cursor was on the incorrect side. We calculated this measure for every trial and then averaged across all trials to obtain the individual measure. The results are robust to using other similar measures, for instance, a simple binary variable indicating whether the cursor was on the correct (left or right) side of the screen when it crossed the invisible line.

### Reporting summary

Further information on research design is available in the Nature Research Reporting Summary linked to this article.

## Supplementary information

Supplementary Information

Reporting Summary

## Data Availability

The data sets generated during and analyzed for the current study are publicly available in the OSF repository at: https://osf.io/v54nz/. The source data underlying Figs. 2–4 and Supplementary Figs. 1-2 are provided as a Source Data file.

## Source data

Source Data

## Code availability

The code reproducing the analysis is publicly available in the OSF repository at: https://osf.io/v54nz/.
