# Supplementary material for: Mouse tracking reveals structure knowledge in the absence of model-based choice
Source: Nat Commun. 2020 Apr 20;11:1893. doi: 10.1038/s41467-020-15696-w (PMC7170897; doi:10.1038/s41467-020-15696-w)
Supplement: Supplementary file 1 — Supplementary Information [file 41467_2020_15696_MOESM1_ESM.pdf]

# Supplementary Information

## Mouse Tracking Reveals Structure Knowledge in the Absence of Model-Based Choice

Arkady Konovalov & Ian Krajbich

**Supplementary Note 1**

Specific descriptions of the patterns used in the deterministic condition:

- Pattern 1: to get the same bottom fractal in each trial, the subject has to choose the left top fractal twice, then the right top fractal twice, etc. Otherwise the bottom fractal switches between trials.

- Pattern 2: to get the same bottom fractal in each trial, the subject has to avoid choosing the same top fractal 3 or more times in a row. If the subject does choose the same top fractal 3 times in a row then the bottom fractal switches and continues to switch each trial until the subject chooses a different top fractal.

- Pattern 3: to get the same bottom fractal in each trial, the subject has to constantly alternate between the top fractals. To switch from one bottom fractal to the other, the subject simply needs to choose the same top fractal twice.

- Pattern 4: to get one of the bottom fractals in each trial, the subject has to choose the same top fractal as in the previous trial. To get the other bottom fractal, the subject has to choose a different top fractal than in the previous trial.

**Supplementary Note 2**

In addition to the model presented in the main text, we also tested several variants. All of these models shared a common TD($\lambda$) component as follows.

The value of the chosen bottom fractal is updated using the model-free Rescorla-Wagner rule:

$$v_t = v_{t-1} + \alpha(r_t - v_{t-1}), \quad (1)$$

where $v_t$ is the estimated value of the bottom fractal on trial $t$, $r_t$ is the actual reward received on trial $t$, and $\alpha$ is the learning rate. The model-free Q-value of each of the chosen top fractals is updated using a TD mechanism:

$$q_t^{MF} = (1-\alpha) \cdot q_{t-1}^{MF} + \alpha \cdot (v_{t-1} - \lambda \cdot (r - v_{t-1})), \quad (2)$$

where $v_t$ is the value of the bottom fractal on trial $t$, and $r_t$ is the reward on trial $t$.

We also assigned a model-based Q-value to the top fractal choice. In the stochastic condition, this value was equal to the expected value of the choice: $q_t^{MB} = p_L v_t^L + p_R v_t^R$, where $p_i$ represent the true probabilities of getting to the left (L) or right (R) bottom fractal after choosing the specific option, and $v_t^i$ are the cached model-free values of the bottom fractals. In the deterministic condition, since the coming bottom fractal was uniquely defined from the underlying pattern based on the previous history of top fractal choices, the model-based value of each top fractal was simply equal to the cached value of the bottom fractal that would appear (according to the pattern) if that top fractal was chosen.

In the final step of the model, we used the standard hybrid combination of the model-free and model-based Q-values:

$$q_t^{HYB} = w \cdot q_t^{MB} + (1-w) \cdot q_t^{MF}, \quad (3)$$

where $w$ is the weight index reflecting the degree of model-based behavior.

We used the difference of Q-values for the top fractals as an input in a standard logit choice model (softmax) with a temperature parameter $\beta$.

All the models we tested were variants of this general model:

- TD(1) model assumes $\lambda = 1$;
- TD(0) model assumes $\lambda = 0$;
- Model-free model assumes w = 0;
- The perseverance model assumes that the softmax function has an additional bonus $p$ (free parameter) that is added to the value of the option chosen on the previous trial. This bonus for the previous choice only enters into the logit and does not affect the Q-values of the options.

**Supplementary Figures**

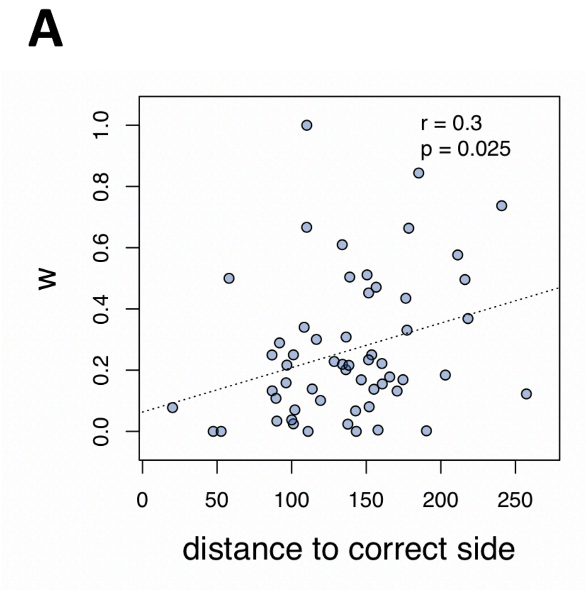


**Supplementary Figure 1.** (A-B) Correlation between the model-based *w* and mouse-tracking measure of learning in stochastic (A) and deterministic (B) conditions.

All correlation plots show Pearson correlations, each individual point is one subject, and dotted lines indicate linear regression fits. Source data are provided as a Source Data file.

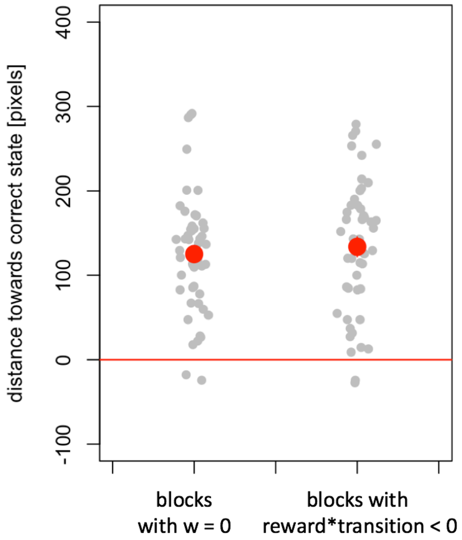


**Supplementary Figure 2.** Individual estimates of structure knowledge from mouse-tracking. Each gray dot is a subject, and the red dot shows the mean distance of the mouse cursor towards the correct (most likely) state, significantly different from 0 at $p = 10^{-10}$ (two-sided t-test; $t(49) = 12.6$, and $t(46) = 11.3$).

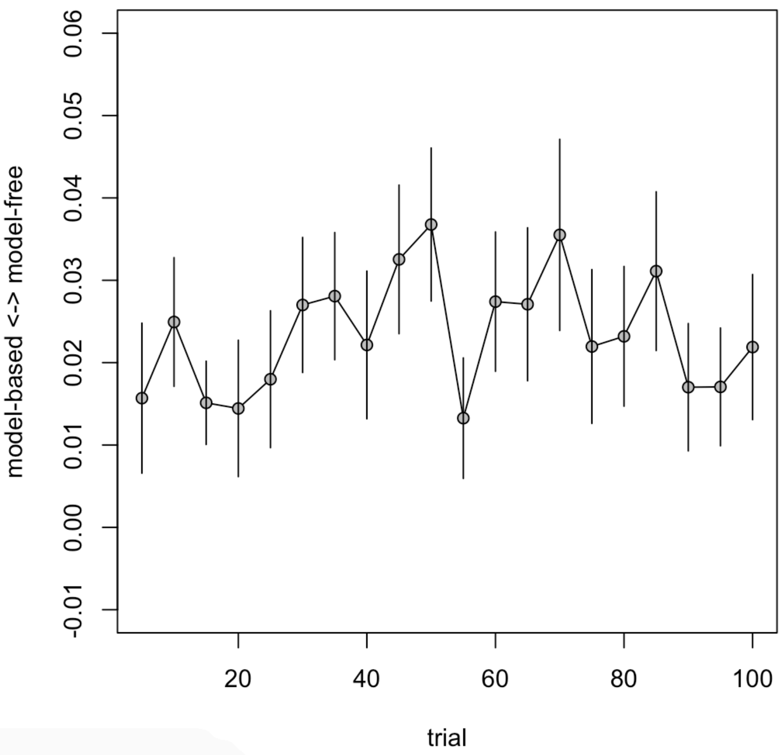


**Supplementary Figure 3**. Evolution of model-based regression coefficient measure (Fig. 2A-C and Fig. 3B in the main text), averaged across subjects, over 100 trials of the block, across all conditions of the stochastic task. To obtain each point, we regressed p(stay) on reward in the rare transitions (run every 5 trials, across subjects, using a mixed-effects regression with subjects and transition probabilities as random effects) and plotted the average coefficients across trials. There was no significant trend in these coefficients across trials (linear regression of the coefficient on the trial number, $p = 0.46$). Error bars denote s.e.m. at the subject level ($N = 57$).

**A**

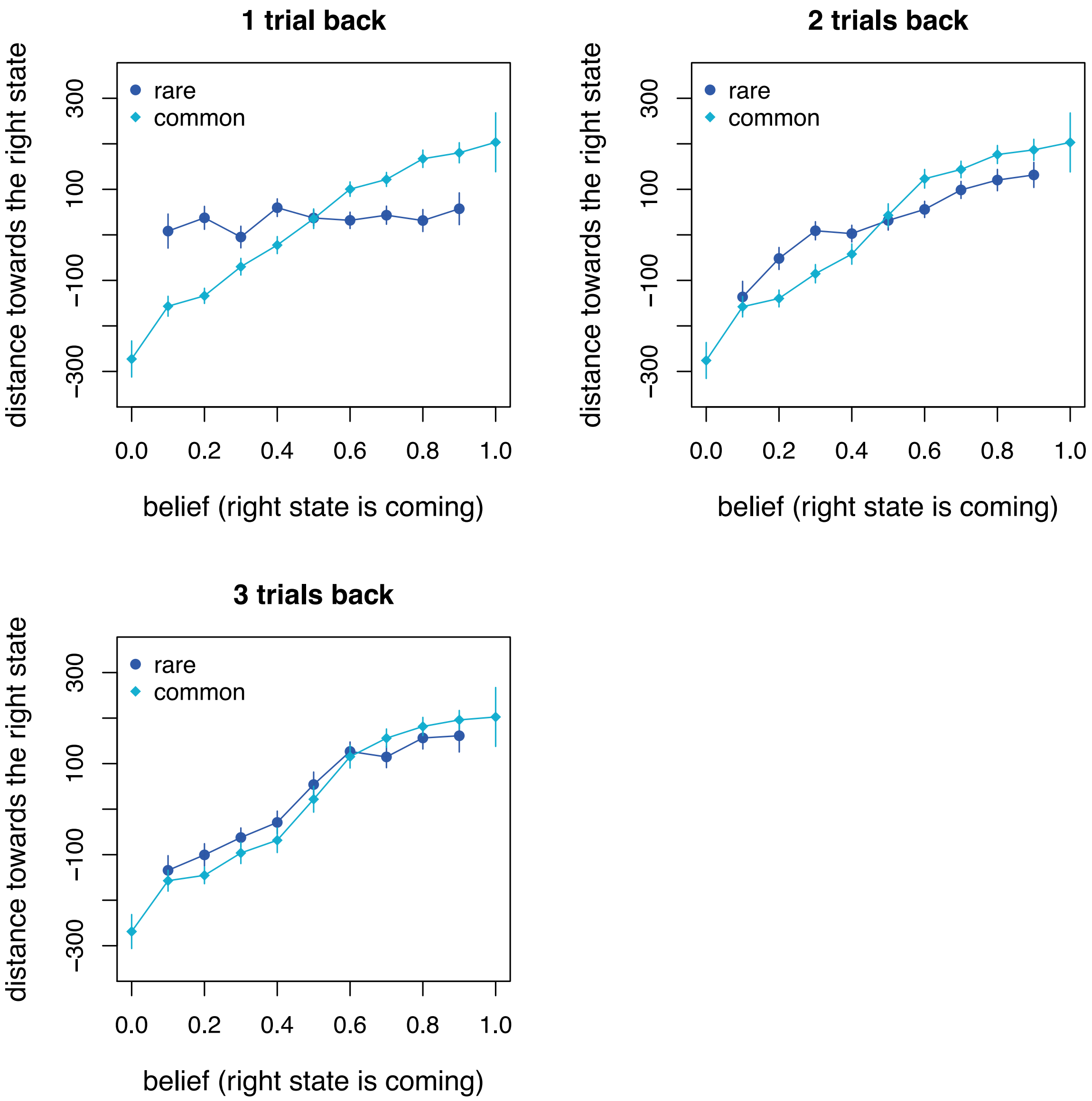


**Supplementary Figure 4A.** Mouse movements following a rare transition. (A) Mouse-tracking measure of structure knowledge in the stochastic task (distance toward the right-hand state) as a function of posterior Bayesian belief that the right-hand state is coming on the current trial, split by common and rare transitions, and the timing of the rare transition (previous trial, 2 trials back, 3 trials back).

# B

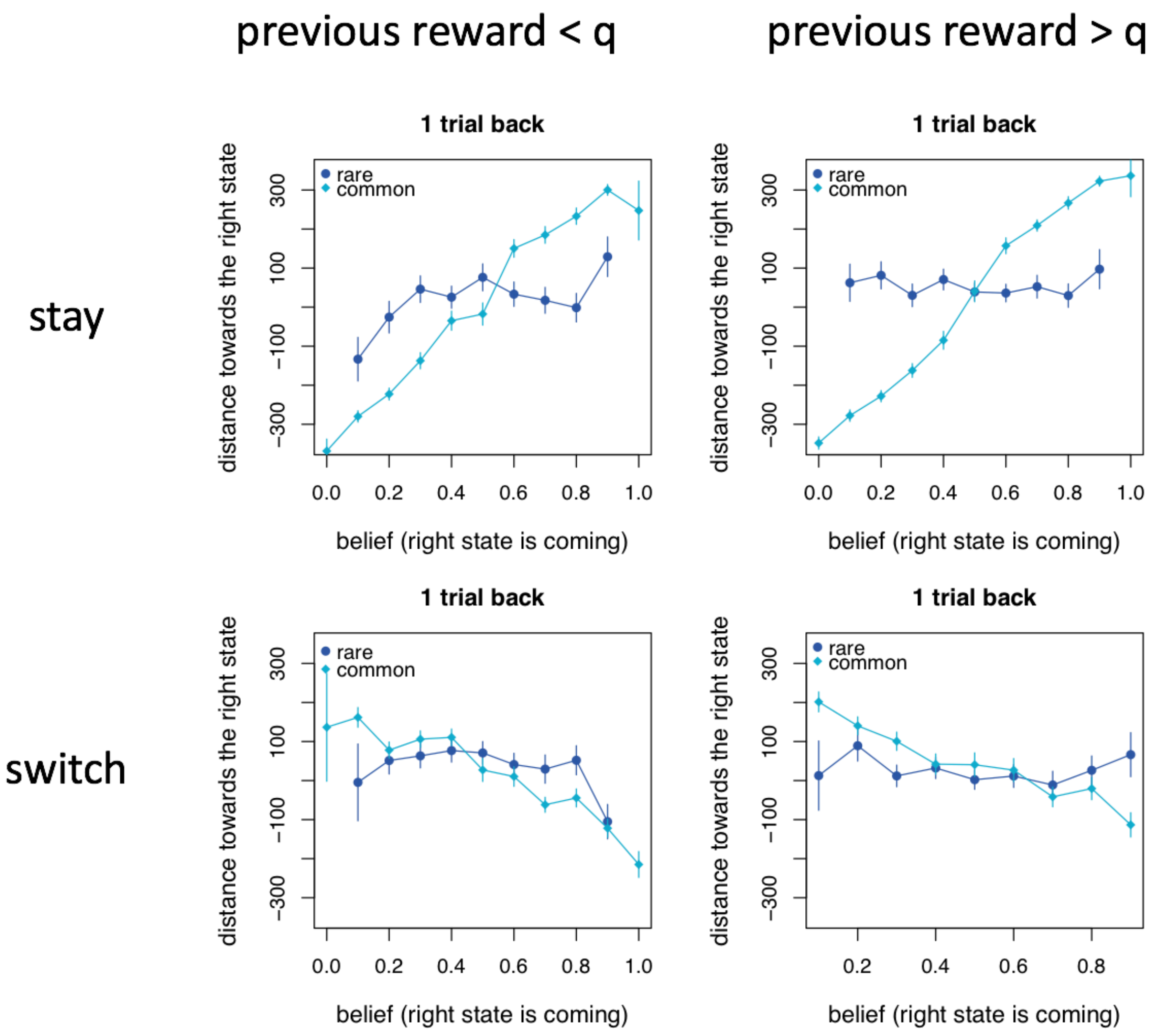


**Supplementary Figure 4B**. (B) The 1-trial-back figure, further split by previous trial reward (<50 and >=50), and by the current-trial decision (stay vs switch).
It is as if, after a rare but rewarding transition, subjects seem to attempt a replay of the previous trial, choosing the same first-stage state and expecting the same (rare) transition. In the case of switch, the mouse movements are either uninformative or in the wrong direction, suggesting some lag between shifts in strategy and mouse movements. Error bars denote s.e.m. at the subject level (N = 57).

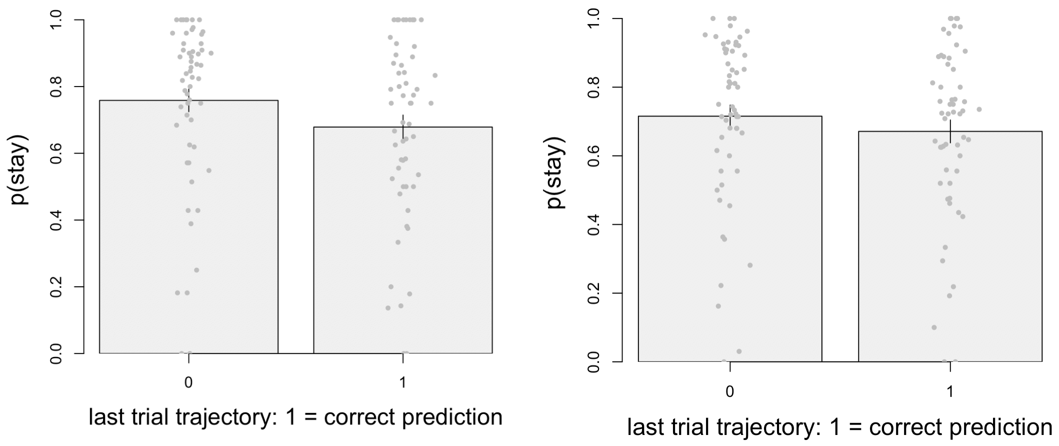


**Supplementary Figure 5**. Probability to stay with the same first-stage choice conditional on the previous trial's mouse trajectory, where 1 denotes a correct mouse trajectory. The analysis focuses on data from the stochastic task, on trials following a rare transition that yielded a high reward (> 50 in the left panel, > than the Q-value of the second-stage fractal in the right panel). Subjects were more likely to switch after a correct mouse trajectory than after an incorrect trajectory. However, in both cases subjects were more likely to stay than to switch, indicating predominantly model-free behavior. Error bars denote s.e.m. at the subject level (N = 57). The gray dots denote individual subjects.

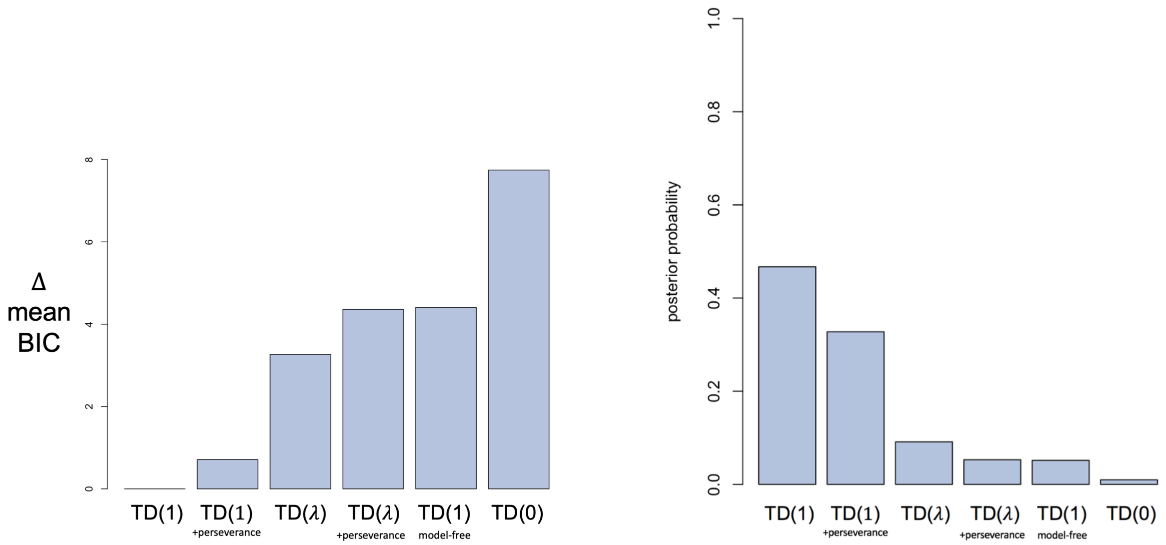


**Supplementary Figure 6**. Computational model comparison. Average BIC values (left panel) and posterior model probability (right panel) across subjects for 6 competing models: the model used in the paper (no perseverance, eligibility trace $\lambda = 1$), a TD(1) model with a perseverance parameter, a model with a flexible eligibility trace $\lambda$, a model with both the perseverance parameter and a flexible $\lambda$, a simple model-free learner ($w = 0$), and a model including TD(0). Lower BIC denotes better model fit. The values are shown subtracting BIC of the best-fitting model (TD(1)). For the detailed description of the models see Supplementary Note 2. Source data are provided as a Source Data file.

## A. TD(1) with perseverance:

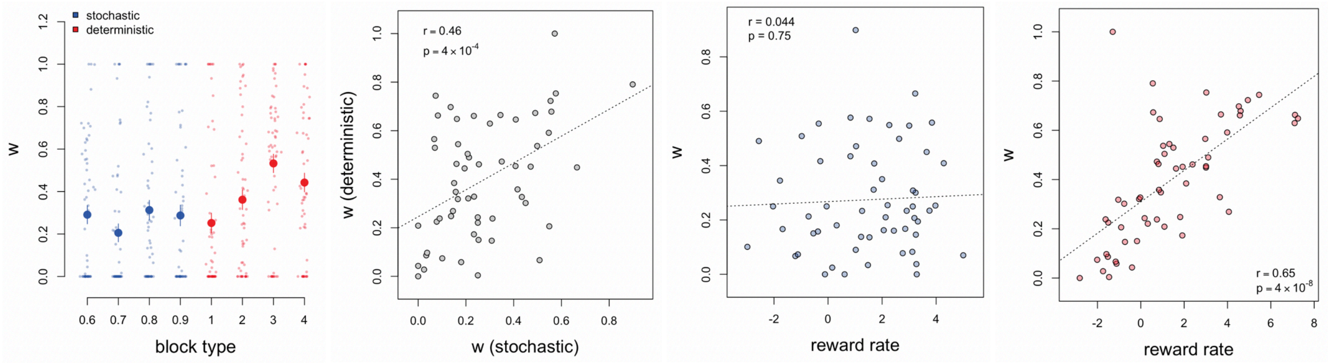


## B. TD($\lambda$):

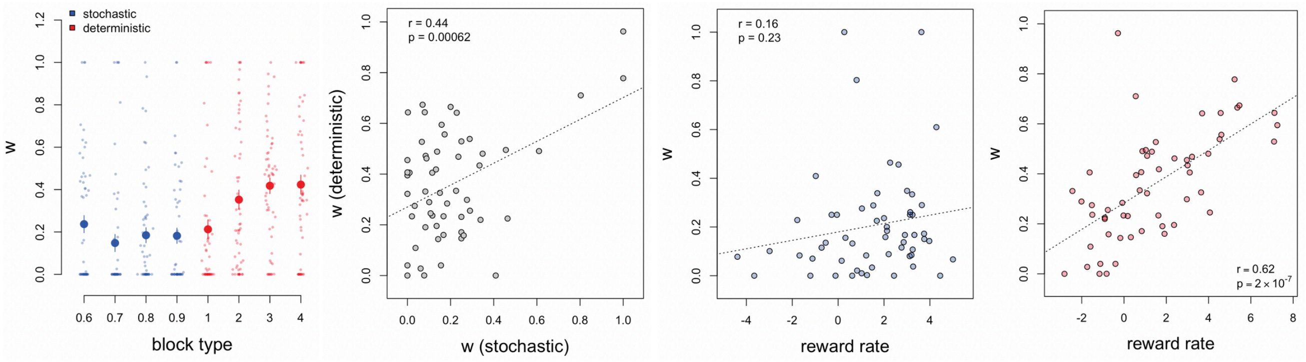


## C. TD($\lambda$) with perseverance

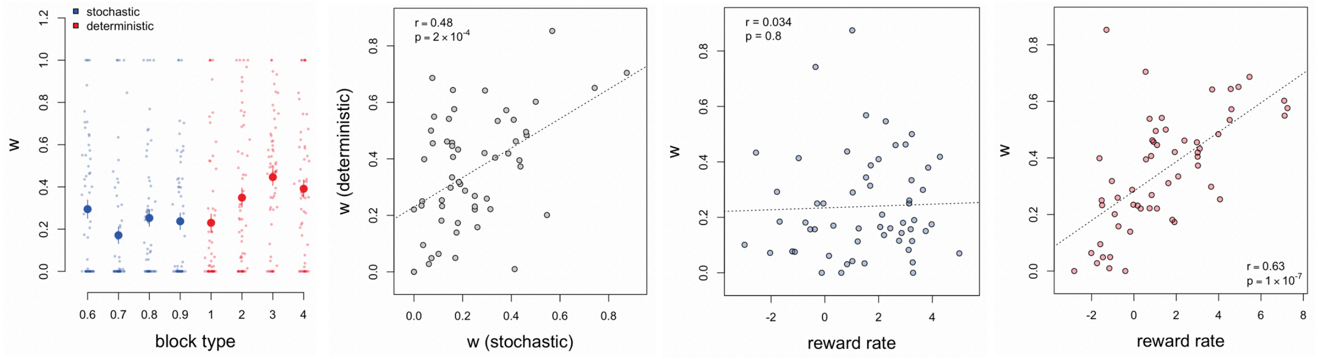


**Supplementary Figure 7**. Main results related to the model-based weight measure *w* using alternative models (corresponding to Figure 2D, 2E, 2H, and 2I in the main text). Error bars denote s.e.m. at the subject level (N = 57).
