# Supplementary material for: Mouse tracking reveals structure knowledge in the absence of model-based choice
Source: Nat Commun. 2020 Apr 20;11:1893. doi: 10.1038/s41467-020-15696-w (PMC7170897; doi:10.1038/s41467-020-15696-w)
Supplement: Supplementary file 2 — Reporting Summary [file 41467_2020_15696_MOESM2_ESM.pdf]

nature research

Corresponding author(s): Ian Krajbich

Last updated by author(s): 16/03/2020

Reporting Summary

Nature Research wishes to improve the reproducibility of the work that we publish. This form provides structure for consistency and transparency in reporting. For further information on Nature Research policies, see Authors & Referees and the Editorial Policy Checklist .

Statistics

For all statistical analyses, confirm that the following items are present in the figure legend, table legend, main text, or Methods section.

n/a	Confirmed	
☐	☒	The exact sample size (*n*) for each experimental group/condition, given as a discrete number and unit of measurement
☐	☒	A statement on whether measurements were taken from distinct samples or whether the same sample was measured repeatedly
☐	☒	The statistical test(s) used AND whether they are one- or two-sided<br>*Only common tests should be described solely by name; describe more complex techniques in the Methods section.*
☐	☒	A description of all covariates tested
☐	☒	A description of any assumptions or corrections, such as tests of normality and adjustment for multiple comparisons
☐	☒	A full description of the statistical parameters including central tendency (e.g. means) or other basic estimates (e.g. regression coefficient) AND variation (e.g. standard deviation) or associated estimates of uncertainty (e.g. confidence intervals)
☐	☒	For null hypothesis testing, the test statistic (e.g. *F*, *t*, *r*) with confidence intervals, effect sizes, degrees of freedom and *P* value noted<br>*Give P values as exact values whenever suitable.*
☒	☐	For Bayesian analysis, information on the choice of priors and Markov chain Monte Carlo settings
☒	☐	For hierarchical and complex designs, identification of the appropriate level for tests and full reporting of outcomes
☐	☒	Estimates of effect sizes (e.g. Cohen's *d*, Pearson's *r*), indicating how they were calculated

Our web collection on statistics for biologists contains articles on many of the points above.

Software and code

Policy information about availability of computer code

Data collection	MATLAB 2017, Psychtoolbox 3, custom experiment code (publicly available at https://osf.io/v54nz/?view_only=f44b7f491325478da33b68e591cb57a9)
Data analysis	R 3.5.2, R packages lme4 1.1-21, lmerTest 3.1-0, custom model code (publicly available at https://osf.io/v54nz/?view_only=f44b7f491325478da33b68e591cb57a9)

For manuscripts utilizing custom algorithms or software that are central to the research but not yet described in published literature, software must be made available to editors/reviewers. We strongly encourage code deposition in a community repository (e.g. GitHub). See the Nature Research guidelines for submitting code & software for further information.

Data

Policy information about availability of data

All manuscripts must include a data availability statement. This statement should provide the following information, where applicable:

- Accession codes, unique identifiers, or web links for publicly available datasets
- A list of figures that have associated raw data
- A description of any restrictions on data availability

The data sets generated during and analyzed for the current study are publicly available in the OSF repository at: https://osf.io/v54nz/?view_only=f44b7f491325478da33b68e591cb57a9. The source data underlying Figs 2-4 and Supplementary Figs 1-2 are provided as a Source Data file.

Field-specific reporting

Please select the one below that is the best fit for your research. If you are not sure, read the appropriate sections before making your selection.

☐ Life sciences ☒ Behavioural & social sciences ☐ Ecological, evolutionary & environmental sciences

For a reference copy of the document with all sections, see nature.com/documents/nr-reporting-summary-flat.pdf

Behavioural & social sciences study design

All studies must disclose on these points even when the disclosure is negative.

Study description	The study uses a novel mouse-tracking method to estimate subjective beliefs in a reinforcement learning task in a human subjects experiment. The study demonstrates that individuals learn task structure, but do not necessarily use it to make choices.
Research sample	We recruited 58 adult subjects (20 female, age 18-22) from the Department of Economics undergraduate subject pool at the Ohio State University, which includes students from across the university and is thus a representative sample of college students . We based the sample size on the previous literature and effect estimates from pilot data (see sampling strategy below).
Sampling strategy	We determined the target sample size aiming to estimate the significant correlation between an individual mouse-tracking measure and the model-based index on the individual level assuming the Pearson correlation coefficient of 0.5, 0.01 significance level, and 90% power, which resulted in a minimal sample of 52 subjects. We invited 60 subjects for two 30-person sessions.
Data collection	The study used a standard experimental economics laboratory, where the subjects could not see each others' screens, and participated in the experiment using computer terminals. The first author was present during data collection.
Timing	The data was collected in 4 sessions conducted in May - June 2017.
Data exclusions	Out of 58 subjects who participated, we excluded one subject who failed to complete the task in reasonable time, leaving 57 subjects for all the analyses.
Non-participation	No participants declined to participate.
Randomization	The study did not use across-subject randomization.

Reporting for specific materials, systems and methods

We require information from authors about some types of materials, experimental systems and methods used in many studies. Here, indicate whether each material, system or method listed is relevant to your study. If you are not sure if a list item applies to your research, read the appropriate section before selecting a response.

Materials & experimental systems

n/a	Involved in the study	
☒	☐	Antibodies
☒	☐	Eukaryotic cell lines
☒	☐	Palaeontology
☒	☐	Animals and other organisms
☐	☒	Human research participants
☒	☐	Clinical data

Methods

n/a	Involved in the study	
☒	☐	ChIP-seq
☒	☐	Flow cytometry
☒	☐	MRI-based neuroimaging

Human research participants

Policy information about studies involving human research participants

Population characteristics	We recruited 58 adult subjects (20 female, age 18-22) from the Department of Economics undergraduate subject pool at the Ohio State University, which includes students from across the university and is thus a representative sample of college students .
Recruitment	The participants were volunteers recruited through the Department of Economics undergraduate subject pool at the Ohio State University. The study sample was restricted to college students.
Ethics oversight	The Ohio State University Internal Review Board.

Note that full information on the approval of the study protocol must also be provided in the manuscript.
