# Supplementary material for: Mouse tracking reveals structure knowledge in the absence of model-based choice
Source: Nat Commun. 2020 Apr 20;11:1893. doi: 10.1038/s41467-020-15696-w (PMC7170897; doi:10.1038/s41467-020-15696-w)
Supplement: Supplementary file 3 — Source Data [file 41467_2020_15696_MOESM3_ESM.zip › readme.rtf]

Contents:	•	MT_fit_functions.R: functions for model fits	•	MT_plot_functions.R: custom plotting functions	•	MT_sim_data.R: script that simulates the model	•	MT_model_fit.R: this script takes MT_data.Rdata and fits the model to the data, and produces MT_data_fitted.RData (takes about an two hours depending on the computer)	•	MT_analysis.R: this script takes MT_data_fitted.RData and produces figures and analyses in the paper (see included output folder)	•	output: figures in the paper in PDF formatRequires:RTested under:macOS Sierra 10.12.6R 3.5.2.No installation required. Main analysis script is MT_analysis.R.Distributed under GNU General Public License, version 3.0.
